# Wearable Detection of Trunk Flexions: Capacitive Elastomeric Sensors Compared to Inertial Sensors

**DOI:** 10.3390/s21165453

**Published:** 2021-08-12

**Authors:** Gabriele Frediani, Leonardo Bocchi, Federica Vannetti, Giovanni Zonfrillo, Federico Carpi

**Affiliations:** 1 Department of Industrial Engineering, University of Florence, 50121 Florence, Italy; gabriele.frediani@unifi.it (G.F.); giovanni.zonfrillo@unifi.it (G.Z.); 2 Department of Information Engineering, University of Florence, 50121 Florence, Italy; leonardo.bocchi@unifi.it; 3 IRCCS Fondazione don Carlo Gnocchi ONLUS, 50143 Florence, Italy; fvannetti@dongnocchi.it

**Keywords:** capacitive, elastomer, flexion, sensor, wearable, wireless

## Abstract

Continuous monitoring of flexions of the trunk via wearable sensors could help various types of workers to reduce risks associated with incorrect postures and movements. Stretchable piezo-capacitive elastomeric sensors based on dielectric elastomers have recently been described as a wearable, lightweight and cost-effective technology to monitor human kinematics. Their stretching causes an increase of capacitance, which can be related to angular movements. Here, we describe a wearable wireless system to detect flexions of the trunk, based on such sensors. In particular, we present: (i) a comparison of different calibration strategies for the capacitive sensors, using either an accelerometer or a gyroscope as an inclinometer; (ii) a comparison of the capacitive sensors’ performance with those of the accelerometer and gyroscope; to that aim, the three types of sensors were evaluated relative to stereophotogrammetry. Compared to the gyroscope, the capacitive sensors showed a higher accuracy. Compared to the accelerometer, their performance was lower when used as quasi-static inclinometers but also higher in case of highly dynamic accelerations. This makes the capacitive sensors attractive as a complementary, rather than alternative, technology to inertial sensors.

## 1. Introduction

Many types of workers have to deal with tiring, incorrect, and even risky body postures and movements, which involve excessive and/or repeated flexions of the trunk while accomplishing duties. This can cause pain and increase the risk of musculoskeletal injuries, such that the trunk’s flexion is a key variable to assess risks associated with incorrect and dangerous postures in occupational health management [1,2,3,4]. Therefore, a continuous monitoring of body flexions could help to adopt corrective measures, especially to prevent injuries.

The gold standard to measure human body kinematics is represented by stereophotogrammetry, which uses external cameras to track the position of markers arranged on the subject [5]. Although this technique has a high accuracy, it is not always applicable to monitor workers. Indeed, not only does it require bulky, complex-to-use, and expensive equipment, but it also requires the subject to be confined within an empty space, so as to enable continuous tracking by the cameras.

Therefore, in order to increase the ease of use and versatility, the ideal sensor should be fully wearable.

Among conventional wearable technologies available to monitor body motions [6], the most sensitive and most used are inertial measurement units (IMUs), electrogoniometers, and electromagnetic sensors.

IMUs combine tri-axial gyroscopes, accelerometers, and magnetometers in one small device. Gyroscopes and accelerometers are typically affected by errors, respectively due to drift (resulting from the integration of an angular velocity) and sudden accelerations/decelerations, such that compensations between them, frequently also using the co-located magnetometer, are needed [7,8,9]. Such a sensory fusion complicates the monitoring algorithms and, especially, the use of the magnetometer makes the units sensitive to electromagnetic interference. Moreover, the output of such systems is often complemented with biomechanical models, so as to resolve uncertainties and further improve the accuracy [7], leading to increased complexity and computational cost.

Electrogoniometers use strain gages, which show ease of use and low cost. However, as they need to be placed over joints, their limited flexibility and relative size tend to interfere with the joint’s kinematics and cause discomfort [10,11]. Notably, attempts to overcome these drawbacks are ongoing with new types of electrogoniometers made of electro-conductive yarns (knitted piezo-resistive fabrics) [12,13].

Electromagnetic sensing uses externally generated magnetic fields to achieve a three-dimensional localization of wearable receiving coils relative to the external emitters, with potentially high accuracy. However, this technique has a limited workspace (due to the spatial decay of the field strength) and is susceptible to errors caused by electromagnetic interference [14,15].

Less conventional wearable sensing strategies include stretchable piezo-resistive sensors, made of conductive elastomers. They are stretch sensors, consisting of planar deformable resistors, which increase their electrical resistance upon stretching. This is a highly versatile technology, which advantageously enables deep integration, within garments, of large arrays of distributed, lightweight, comfortable, easy-to-use, and cost-effective sensors [16,17]. However, their output can be affected by significant viscoelastic creep and is dependent on ambient temperature and humidity, which change the material’s resistivity [16,17].

So, according to this state of the art, it is currently not possible to combine into a single system all the desirable properties of an ideal wearable sensor, namely high accuracy, stable response, large workspace, compact size, lightweight, comfort, ease of use, and low cost.

In recent times, the kinematics of human movements has started to be investigated also with stretchable piezo-capacitive sensors made of dielectric elastomers. As for the stretchable piezo-resistive sensors mentioned above, they too are elastomeric stretch sensors. However, they consist of planar deformable capacitors, which increase their electrical capacitance upon stretching [18]. They are obtained from an elastomeric insulating layer (e.g., made of a silicone rubber) sandwiched between two layers of a deformable electrode material (e.g., a carbon black-loaded silicone rubber), so as to assemble a stretchable capacitor. By arranging a set of these sensors such that they are stretched by specific postures and movements, following a calibration it is possible to relate occurring variations of capacitance to thee motions of body segments, as shown by several studies [19,20,21,22,23,24,25,26,27,28].

Recently, some of us presented a low-cost wearable concept to monitor movements of the trunk, using elastomeric capacitive sensors in combination with modified shoulder straps [29]. The latter were intended to make the system easily wearable onto or under common clothes, so as to avoid less practical/stable, uncomfortable or more expensive solutions, such as sticking the sensors onto the skin [22,23,24,25,27] or creating dedicated elastic garments that integrate printed sensors [26].

Here, we present a full implementation of this system as a simple inclinometer and, especially, we address two critical issues:

(i) The implementation of a calibration strategy that avoids the limitations of typical approaches reported in the literature, such as measuring the angles with bulky, unpractical, and/or expensive external equipment (e.g., stereophotogrammetry [19,20,21,22,23,24,25]), or using interpolations of data captured from reference poses (which enables pose recognitions but does not provide accurate measurements of angles [26,27,28]).
(ii) A systematic comparison of the performance of this capacitive technology with that of conventional inertial sensors (accelerometer and gyroscope of an IMU); to that aim, the three sensing technologies were evaluated relative to stereophotogrammetry (as the non-wearable standard system).

## 2. Materials and Methods

### 2.1. The Wearable System

**Figure 1:**
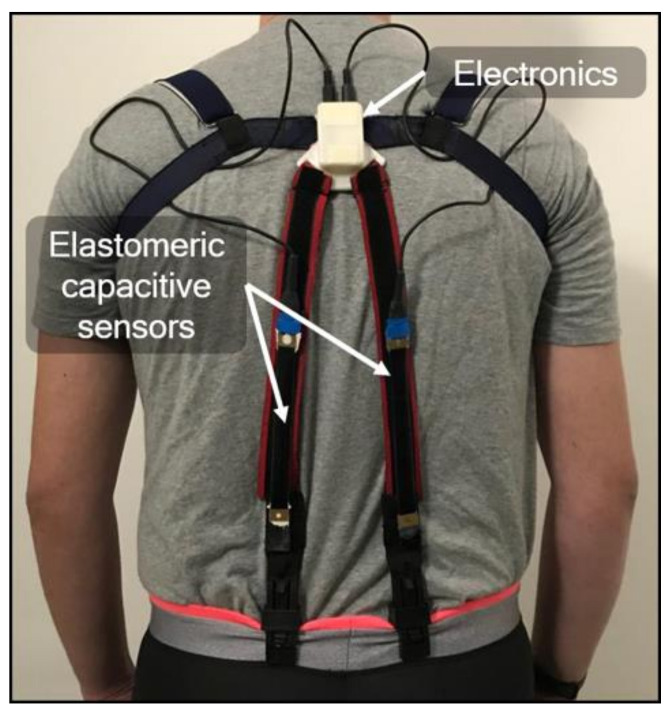
Picture of the assembled wearable system, based on two capacitive stretch sensors.

The system was conceived to detect pure flexions in the user’s sagittal plane. To this end, conventional shoulder straps made of an elastic fabric were modified, so as to integrate in their lower section two rectangular silicone-based capacitive stretch sensors (Courtesy of Parker Hannifin, Mayfield Heights, OH, USA), as shown in Figure 1. The sensors had at rest (i.e., prior to their pre-stretching, induced while securing the straps) a length of 100 mm, a width of 15 mm, and a total thickness of 1 mm (including the insulating coatings of the electrodes). Their sensitivity, expressed as the ratio between the percentage variation of capacitance and the percentage variation of length (strain), was ~1.

The sensors’ upper ends were attached to a plastic box, which contained wireless electronics and was located between the shoulder blades, whilst the lower ends were clamped to the user’s trousers (Figure 1).

Each volunteer involved in this study (as detailed below) was asked to wear the same type of trousers (gym pants), consisting of an elastic textile, adherent to the body. They were used to minimize the risk of unpredictable displacements, which could change the sensors’ stretching for analogous flexions of any given user.

The arrangement of the sensors was such that they were not parallel to the spine (Figure 1). This configuration, as compared to a possible alternative use of a single sensor aligned with the spine, was motivated by the need to minimize the effect of unintentional asymmetric movements, especially in terms of torsions and lateral bending. In order to limit inaccuracies due to such unintended movements, the two capacitance values were averaged (as described in the following).

### 2.2. Wireless Electronics

The two capacitances were continuously measured by wireless electronics, which combined a Bluno Beetle board (DFRobot, Beijing, China), equipped with a Bluetooth 4.0 module, and an IMU (MPU-6050, TDK InvenSense, New York, NY, USA), equipped with a three-axial accelerometer and a three-axial gyroscope. A LiPo battery with a capacity of 400 mAh ensured continuous operation for ~12 h.

The accelerometer and gyroscope were alternatively tested to both calibrate and evaluate the performance of the capacitive sensors (as detailed below).

In order to measure the capacitance of the two elastomeric sensors, the board was programmed with a custom-made algorithm; it continuously estimated the charging time constant *τ* of each capacitor via a resistor *R* in series, such that the capacitance *C* could be obtained as follows:

(1) C=τR

The board was also programmed to obtain angular measurements from the accelerometer and gyroscope (according to the procedures described in the next section).

The simultaneously measured capacitive and angular data were then wirelessly transmitted to an external computer, interfaced to a BlueTooth module. The computer generated (via its sound card) a synchronization signal, used to synchronize the signals received from the wearable system and those acquired by a stereophotogrammetry system (for comparisons).

Each pair of capacitive data was averaged and processed to obtain angular readings, following the calibration procedure described in the next section.

### 2.3. Use of Inertial Sensors as Inclinometers

**Figure 2:**
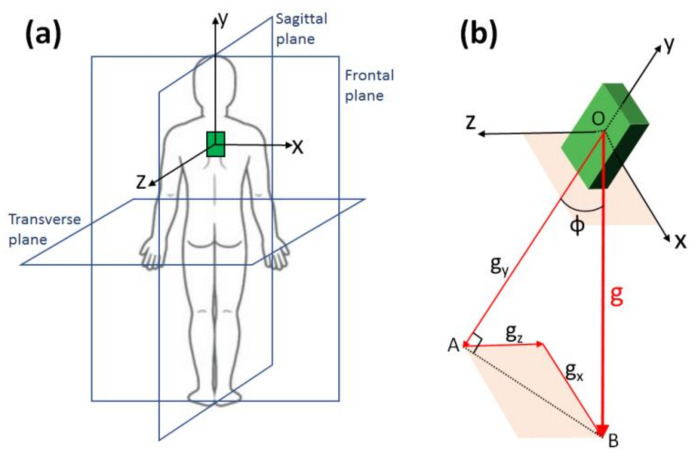
Geometrical description of the use of the IMU as an inclinometer: (**a**) spatial arrangement of the IMU axes; (**b**) definition of a generic inclination of the IMU relative to gravity.

The targeted flexion of the trunk was defined as the angle of the IMU vertical axis relative to the vertical direction (gravity vector) within the sagittal plane (Figure 2a).

Independent measurements of that angle with each inertial sensor of the IMU (used just as an inclinometer) were obtained as follows.

With reference to the IMU axes *x*, *y, z* defined in Figure 2a, a generic inclination angle ϕ of the IMU axis *y* relative to the gravity vector *g* (Figure 2b) can be obtained as follows:

(2) $$\begin{array}{c} ф\;=\arctan\frac{\bar{AB}}{\bar{OA}}=\arctan\frac{\sqrt{g_{x}{}^{2}+g_{z}{}^{2}\;}}{g_{y}}\; \end{array}$$

In the hypothesis that the accelerometer does not experience any acceleration other than gravity, the detected acceleration components *a_x_, a_y_*, and *a_z_* along *x*, *y*, and *z* coincide with the gravity components *g_x_, g_y_*, and *g_z_*; therefore, ϕ is properly obtained from the accelerometer readout, as follows:

(3) $$\begin{array}{c} ф\;=\arctan\frac{\sqrt{a_{x}{}^{2}+a_{z}{}^{2}\;}}{a_{y}} \end{array}$$

For pure flexions within the sagittal plane, the component along *x* is null and so Equation (3) can be simplified to obtain the sagittal inclination ϕ*_sagittal_* (flexion angle) as follows:

(4) $$\begin{array}{c} {ф}_{\mathrm{sagittal}}=\arctan\frac{a_{z}}{a_{y}} \end{array}$$

The gyroscope was used to measure the angular velocity signal *ω_sagittal_*(*t*) within the sagittal plane, i.e., relative to the IMU axis *x* (Figure 2a), and then obtain the flexion angle by integration:

(5) $$\begin{array}{c} {ф}_{\mathrm{sagittal}}=\int_{0}^{t}\omega_{\mathrm{sagittal}}\left(t\right)dt \end{array}$$

### 2.4. Capacitive Sensors’ Calibration with Inertial Sensors: Comparison of Different Strategies

In order to relate the measured average capacitance of the two elastomeric sensors to the flexion angle, a calibration was needed. The calibration was required each time that the system had to be used, even by the same user, so as to comply with a possible variability of the sensors’ positions and pre-stretching that occurred while wearing the shoulder straps and securing them to the trousers.

The calibration had to satisfy the following key requirements: (i) it should be easy to be performed by the user, without assistance from others; (ii) it should not require bulky, unpractical, or expensive external instrumentation; and (iii) it should not require movements of any predefined amplitude, so as to simplify the task and comply with an expected variability in the range of motions that different individuals can cover.

In order to address these needs, the IMU miniaturized sensors were used as inclinometers, not only for performance comparisons, but also for the calibration. This approach assumed that, in a possible future product based on the capacitive sensors, the reading electronics could easily include a compact and cheap IMU. The idea was to employ it only for calibration, avoiding its use for continuous sensing over an extended time and in highly variable conditions, so as not to incur the typical errors and limitations of inertial sensors, as recalled above.

The performance of the accelerometer and the gyroscope for the calibration (according to Equations (4) and (5), respectively) was comparatively investigated as follows.

At the beginning of the calibration, the user was asked to maintain an erect position for 5 s, so as to stabilize the average capacitance signal. A predefined number of samples of the stabilized signal were then averaged and taken as an initial offset, to be subtracted from the subsequent inclination measurements. Then, the user was asked to perform five consecutive flexion-extension cycles, with arbitrary amplitude and a frequency of ~5 cycles/min, as timed by a metronome. This very slow rate was assumed to be sufficiently representative of quasi-static movements, in order to ensure that gravity was the only significant acceleration and so the accelerometer could be used as an inclinometer (Equation (3)).

During the movements, data from the capacitive sensors, accelerometer, and gyroscope were simultaneously recorded. The resulting angle-time signal obtained from each inertial sensor was digitally filtered with an ideal low-pass filter (cut-off frequency of 0.3 Hz). The obtained angular instantaneous values were then plotted as a function of the instantaneous values of the average capacitance, and the data were fitted with a parametric calibration curve, consisting of a second-order polynomial (the second order was found to be sufficient).

In particular, the polynomial fitting was implemented in two alternative ways, which differed according to the considered data set: in a first strategy, a single calibration curve was obtained from the whole range of angles covered by the five flexion-extension cycles; in a second strategy, two calibration curves were obtained by separately grouping the angles corresponding to the five flexions (first phases of the five cycles) and the angles corresponding to the five extensions (second phases of the five cycles). The grouping was performed with a Matlab code, where the instants corresponding to a transition between any flexion and its subsequent extension were automatically identified according to a change of the sign of the signal’s first derivative.

As a result, for a single user, four types of calibrations were achieved. In the following, they are referred to as: ‘calibration with accelerometer and single curve’, ‘calibration with accelerometer and double curve’, ‘calibration with gyroscope and single curve’, and ‘calibration with gyroscope and double curve’.

In order to investigate which calibration strategy offered the highest accuracy, a comparative study was performed on 10 volunteers (7 females and 3 males, aged between 25 and 40 years), as described below.

For each volunteer, the capacitive sensors were firstly calibrated with the four strategies described above. Then, the user was asked to perform an additional eight sets of movements; each set consisted of repeated flexion-extension cycles, having an arbitrary amplitude and a frequency of 10, 15, 20, 25, 30, 35, 40, or 45 cycles/min (faster paces were found too difficult to be followed reliably), as timed by a metronome. Each set was repeated twice. For each frequency, the average capacitance signal was converted into four calibrated angular signals, using the four calibration curves.

**Figure 3:**
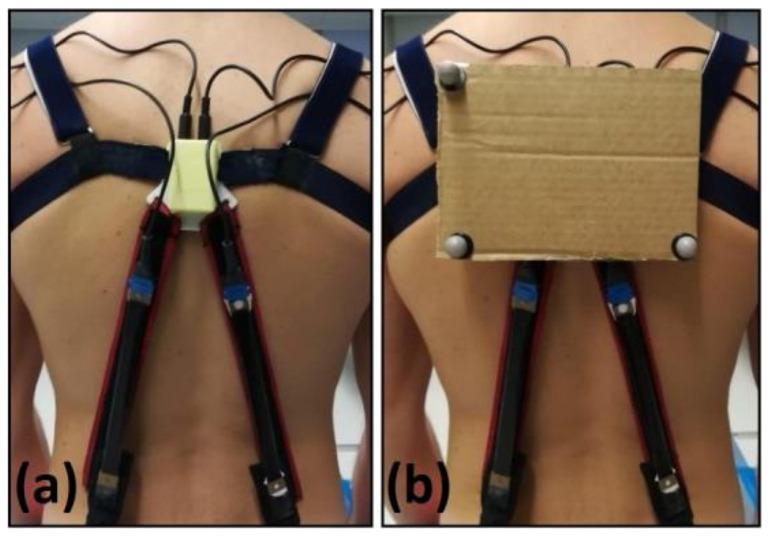
The electronics box (**a**) was equipped with a support for three optical markers (**b**), in order to enable optical tracking of movements of the box via stereophotogrammetry.

In order to evaluate the accuracy of the calibrated signals, each of them was compared with angular measurements simultaneously taken by a conventional stereophotogrammetry system (Smart DX, BTS Bioengineering Srl, Milan, Italy). In particular, in order to enable optical tracking (by the stereophotogrammetry cameras) of the movements of the electronic unit hosting the IMU, and, thus, enable accurate comparisons with its angular measurements, the unit was equipped with a support structure, where three optical markers were arranged, as shown in Figure 3. This unusual way of positioning the markers, avoiding conventional anatomic landmarks, allowed for the highest correlation between the measurements taken by the stereophotogrammetry and those taken by the IMU.

The accuracy of each calibrated signal relative to stereophotogrammetry was quantified by calculating the root mean square error (*RMSE_calibration_*) between the calibrated capacitive signal *S_calib_* and the stereophotogrammetry signal *S_stereo_*:

(6) $$\mathrm{RMS}E_{\mathrm{calibration}}=\sqrt{\frac{\sum_{i}^{n}{\left(\;S_{\mathrm{calib}}{}_{i}-S_{\mathrm{stereo}}{}_{i}\right)}^{2}}{n}}$$

where *S_calib,i_* and *S_stereo,i_* are the *i*-th samples of the signals and *n* is the total number of samples.

The most accurate calibration strategy was selected as the one offering the lowest error.

### 2.5. Performance Comparison between Capacitive Sensors and Inertial Sensors

Following the selection of the most accurate calibration strategy, a comparison between the detection performance (as low-frequency inclinometers) of the calibrated capacitive sensors and those of the accelerometer and gyroscope was performed as follows.

The angular signals measured for each volunteer by the (calibrated) capacitive sensors, the accelerometer, and the gyroscope during the previously described sets of movements were compared with measurements simultaneously taken by the stereophotogrammetry.

The accuracy of each wearable sensing technology relative to stereophotogrammetry was evaluated by calculating, for each set of movements, the root mean square error (*RMSE_sensing_*) between the wearable sensor signal *S_sens_* and the stereophotogrammetry signal *S_stereo_*:

(7) $$\mathrm{RMS}E_{\mathrm{sensing}}=\sqrt{\frac{\sum_{i}^{n}{\left(\;S_{\mathrm{sens}}{}_{i}-S_{\mathrm{stereo}}{}_{i}\right)}^{2}}{n}}$$

where *S_sens,i_* and *S_stereo,i_* are the *i*-th samples of the signals and *n* is the total number of samples.

## 3. Results and discussion

### 3.1. Accuracy of the Different Calibration Strategies

**Figure 4:**
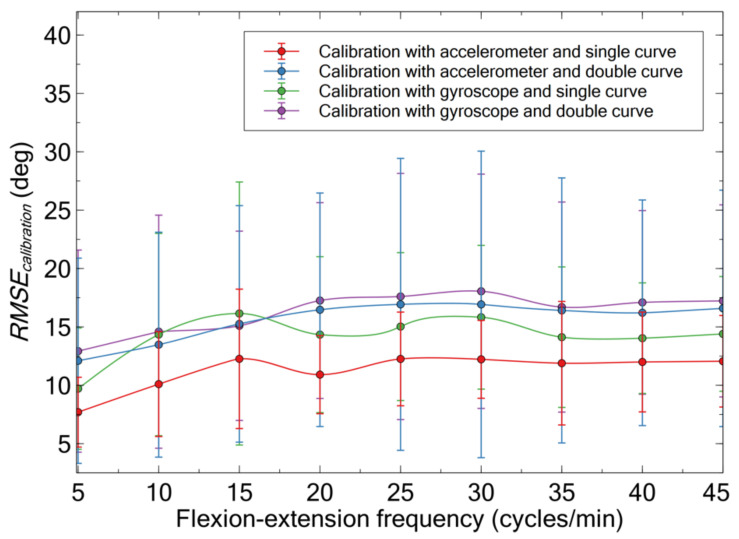
Comparison of the accuracy of the different strategies of calibration of the capacitive sensors, as quantified by the root mean square error between each calibrated signal and the stereophotogrammetry signal, for cyclic flexion-extensions at different frequencies. Each data point is originated by the average among 10 volunteers, considering two repetitions for each volunteer. The error bars represent the standard deviation.

The different accuracy of the described four calibration strategies, as evaluated relative to stereophotogrammetry, can be deduced from the errors presented in Figure 4.

The lowest error was achieved, at each frequency, with the calibration that used the accelerometer and a single curve. Therefore, this calibration strategy was selected and used for the comparative tests, the results of which are presented below.

### 3.2. Accuracy of the Capacitive Sensors Compared to That of the Inertial Sensors

#### 3.2.1. Performance as Low-Frequency Inclinometers

**Figure 5:**
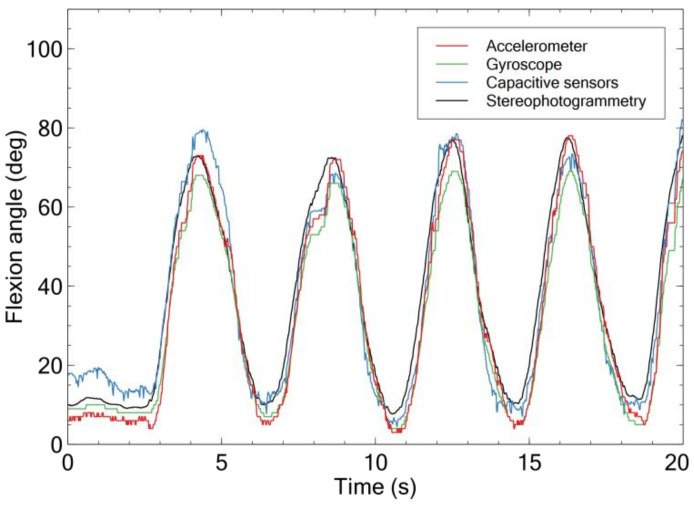
Example of flexion signals simultaneously detected by the calibrated capacitive sensors, accelerometer, gyroscope, and stereophotogrammetry.

Figure 5 shows an example of angular signals simultaneously measured by the (calibrated) capacitive sensors, accelerometer, gyroscope, and stereophotogrammetry.

**Figure 6:**
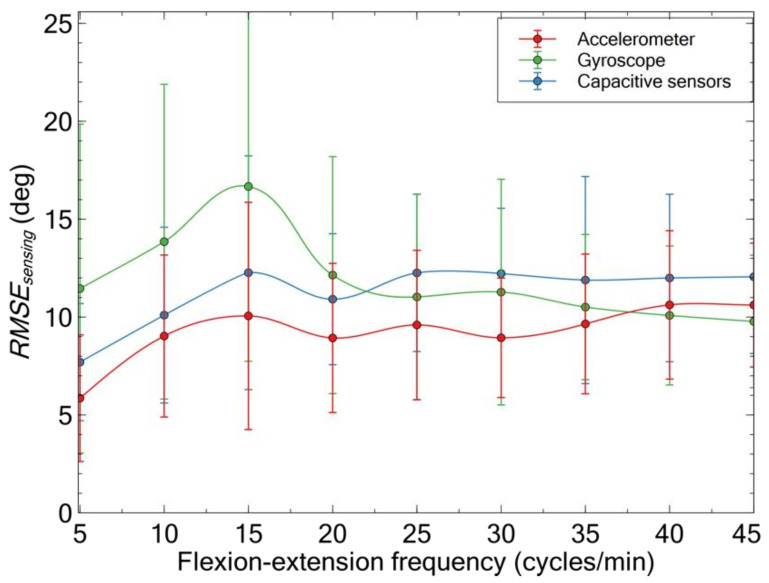
Comparison of the accuracy of the different wearable sensing technologies, as quantified by the root mean square error between the signal measured by each wearable sensor and the stereophotogrammetry signal, for cyclic flexion-extensions at different frequencies. Each data point is originated by the average among 10 volunteers, considering two repetitions for each volunteer. The error bars represent the standard deviation.

For each type of wearable sensor, the motion frequency-dependent error relative to stereophotogrammetry is plotted in Figure 6.

These data show the following evidence. Within the tested frequency range, the root mean square error’s maximum value was ~10 degs for the accelerometer, ~12 degs for the capacitive sensors, and ~17 degs for the gyroscope. The accelerometer showed the highest accuracy nearly across the whole range.

Relative to the accelerometer, the capacitive sensors’ error was systematically higher, up to 20%. Therefore, according to these data, the capacitive sensors would not appear to introduce any advantage as inclinometers.

However, this outcome refers to flexion-extension movements relatively slow, so as to neglect components of acceleration-deceleration and therefore use the accelerometer as an inclinometer (Equation (4)). In reality, the daily activities of various types of workers can generate dynamic accelerations of variable intensity. When the total acceleration significantly deviates from pure gravity, it is reasonable to expect that the accelerometer loses accuracy as an inclinometer. So, in order to compare how the accelerometer and the capacitive sensors behaved in highly dynamic conditions, the tests described in the next section were performed.

#### 3.2.2. Performance against Dynamic Accelerations

**Figure 7:**
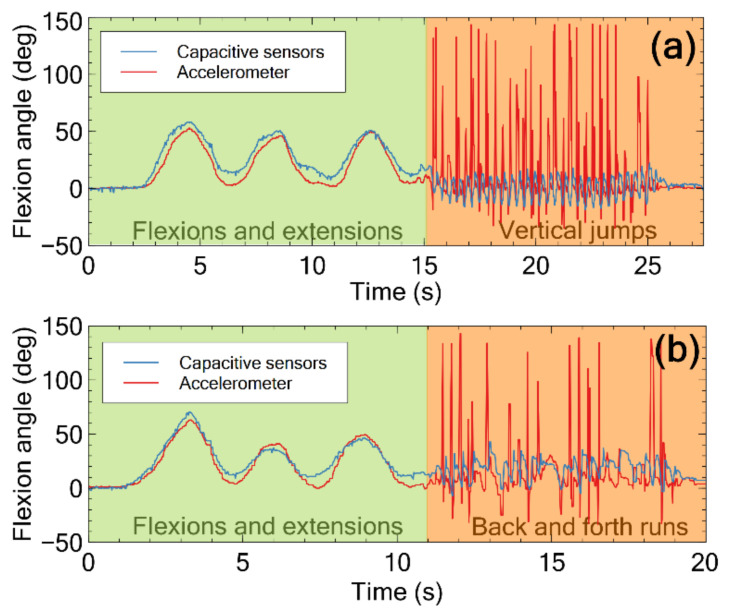
Example of a pair of signals simultaneously detected by the capacitive sensors and the accelerometer during a movement consisting of three low-frequency flexion-extension cycles, followed by a sequence of: (**a**) vertical jumps in place; (**b**) back and forth runs. The peaks detected by the accelerometer lead to an evident angular miss-classification.

Signals obtained from the capacitive sensors and the accelerometer were simultaneously recorded during two types of motion: (i) a sequence of vertical jumps in place; (ii) a sequence of back and forth runs. For each movement, the user was asked to try to keep the trunk as straight as possible, continuously, so as to try to avoid flexion-extension movements. Each dynamic movement was preceded by a sequence of three low-frequency flexion-extension cycles, in order to check that the capacitive sensors and the accelerometer were properly functioning as inclinometers. The results are presented in Figure 7.

As evident, the accelerometer generated large peaks during the jumps and runs, due to dynamic acceleration-decelerations. Those peaks resulted in erroneous estimates of the flexion angle, as revealed by a comparison with the signals gathered by the capacitive sensors. Indeed, the latter were sensitive to the stretch, rather than to the acceleration, thus their dynamic response was not influenced by variations of velocity.

By the way, it is worth noting that even the signals detected by the capacitive sensors did show some (lower) oscillations during the jumps and runs (Figure 7). It is plausible that they were caused by dynamic movements of the trousers’ higher edge (where the sensors were clamped and therefore dynamically stretched), as well as by involuntary flexion-extensions of the trunk.

The lower sensitivity of the capacitive sensors to accelerations, i.e., their higher accuracy in dynamic conditions, overturns the evaluation expressed in the preceding section, based only on the low-frequency tests. Indeed, these additional data suggest that the capacitive sensors do have an advantage over the accelerometer as inclinometers, in that they are less affected by miss-classifications in the presence of accelerations.

**Figure 8:**
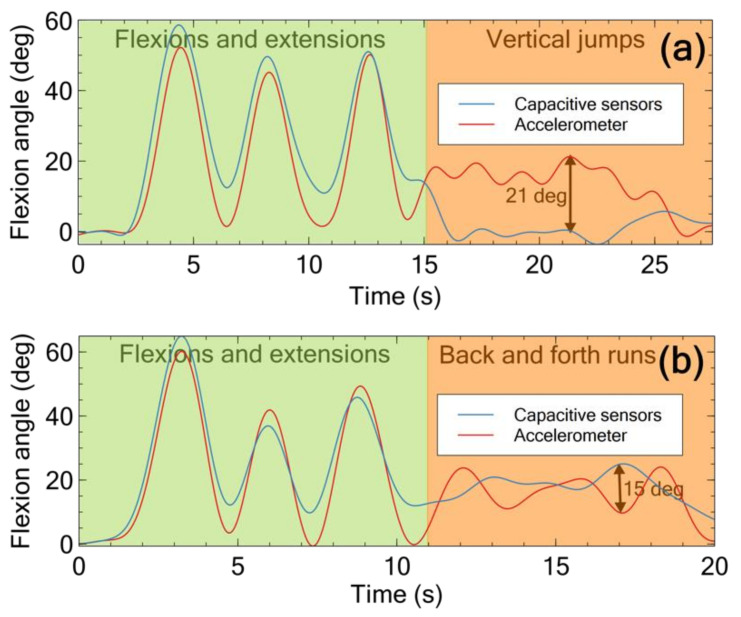
Signals obtained from those presented in the previous figure, following a low-pass digital ideal filtering with a cut-off frequency of 0.5 Hz: (**a**) vertical jumps in place; (**b**) back and forth runs.

Nevertheless, at the same time, it is important to remark that the problem with the accelerometer can be limited with low-pass filtering of its detected signal, so as to reject the high-frequency components related to dynamic accelerations while keeping the low-frequency components related to the actual inclination. As an example, Figure 8 presents the effect of filtering the signals shown in Figure 7, with a cut-off frequency of 0.5 Hz.

As expected, the sharp peaks are removed. However, depending on the type of motion and the concerned accelerations, the residual difference with respect to the capacitive sensors in some cases can still be significant. In particular, Figure 8a shows that, for vertical jumps, the filtered accelerometer’s signal is always larger than the filtered capacitive sensors’ signal and their maximum difference is 21 degs.

Therefore, for some movements with highly dynamic accelerations, even with low-pass filtering the accelerometer can show a higher error relative to the capacitive sensors.

## 4. Conclusions and Future Developments

A wearable system able to monitor flexions of the human upper body, using elastomeric capacitive sensors, was described. The system can easily be worn and used with a simple calibration procedure, which adopts a wearable inertial sensor, to be intentionally used only for the calibration phase.

Different calibration strategies using either only the gyroscope or only the accelerometer of a conventional IMU were proposed and compared, with the best outcomes achieved with the accelerometer.

The gyroscope and the accelerometer were also used to comparatively evaluate the detection performance of the capacitive sensors, by quantifying the accuracy of each technology relative to conventional stereophotogrammetry. Compared to the gyroscope, the capacitive sensors demonstrated a higher accuracy. However, in comparison with the accelerometer, their performance was lower when used as quasi-static inclinometers but also higher in case of highly dynamic accelerations (even after low-pass filtering).

On the other hand, it is evident that the pair of capacitive sensors adopted in this work was much less practical to use than the miniaturized IMU. Moreover, their calibration strategy needed an inertial sensor anyway.

Therefore, by taking into account these pros and cons, in order to monitor a single degree of freedom (as in this study), the capacitive sensors are not attractive as an alternative to inertial sensors but, rather, as a complementary technology to them. Indeed, by detecting flexions via a sensory fusion, it could be possible to ensure a constantly high accuracy, increasing that of the accelerometer in dynamic conditions and that of the capacitive sensors in quasi-static conditions.

However, from a more general standpoint, elastomeric capacitive sensors are expected to offer their greatest potential for the detection of a large number of degrees of freedom. Indeed, the growing complexity introduced by arranging on the body a growing number of IMUs could be avoided with an array of capacitive sensors. In fact, they could be more easily and more conformably distributed over the body, by printing them on garments. The same approach has already been extensively demonstrated for distributed arrays of piezo-resistive elastomeric sensors [16,17] and can straightforwardly be mimicked with piezo-capacitive elastomeric sensors (see, for instance, the prototype glove in Glauser et al. [26] and commercial gloves by StretchSense [30]).

Nevertheless, the use of such arrays of stretchable and distributed capacitive sensors requires a change in the way the function of a wearable sensing system is conceived. Indeed, calibrating them with an array of IMUs, in order to measure a set of angles, would not make sense (excessive complexity and cost, increasing with the array size). Differently, instead of measuring angles, the system should be used to directly recognize poses (postures or gestures), such as a flexed/extended trunk or an open/closed hand, without knowledge of the related angles.

Consistently, angular calibration strategies should be replaced by machine learning algorithms for pose classification. They can efficiently and effectively be used to train, for instance, a neural network, so as to recognize a predefined set of reference poses. Even in this respect, previous experience on distributed piezo-resistive elastomeric sensors [16,17] can be re-applied to piezo-capacitive sensors, as ongoing developments are already demonstrating [26,30].

## Data Availability

The data presented in this study are available on request from the corresponding author. The data are not publicly available due to restrictions on privacy.
